# The causes of injuries sustained at fitness facilities presenting to Victorian emergency departments - identifying the main culprits

**DOI:** 10.1186/s40621-015-0037-4

**Published:** 2015-04-14

**Authors:** Shannon E Gray, Caroline F Finch

**Affiliations:** 1 Monash Injury Research Institute, Monash University, 21 Alliance Lane, Clayton, 3800 Australia; 2 Australian Centre for Research into Injury in Sports and its Prevention, Federation University Australia, Lydiard Street Sth, Ballarat, 3350 Australia

**Keywords:** Fitness centres, Exercise, Weight lifting, Injury, Injury surveillance, Injury coding, Injury prevention

## Abstract

**Background:**

Fitness facilities provide an avenue to engage in physical activity, which is widely encouraged to improve health. However, there is risk of injury. This study aimed to identify the specific causes of injuries sustained at fitness facilities and the activity being participated in, to aid in the development of injury prevention strategies.

**Methods:**

Analysis of routinely collected emergency department case-series data were obtained from July 1999 to June 2013. Fitness activity-related injury cases were identified from narratives of injury events, with narrative information recoded into cause of injury and activity at time of injury categories. Recoded data were then analysed.

**Results:**

Overall, 2,873 cases were identified that specified the exact cause of injury associated with injuries that occurred at fitness facilities. Injuries due to overexertion were most common overall (36.2% of all cases), as well as the main cause of injuries related to general free weight activities (52.6% of this activity) and group exercise classes (35.9%). Crush injuries due to falling weights were common for all free weight activities. Falls and awkward landings were common causes of injuries during group exercise classes (28.5% and 25.8%, respectively). Trips and falls were common throughout facilities, as well as from cardiovascular equipment more specifically.

**Conclusions:**

Detailed information on the causes of injuries allows the development of injury prevention strategies for fitness facilities and fitness activities. Facilities should implement risk management strategies to reduce the risk of injuries in their clientele, based on the identified major causes of injury in this study.

## Background

It is widely accepted that physical activity can enhance overall health and prevent chronic illness (Bull et al. 2004). The fitness industry provides an avenue for people to be physically active and is increasing in popularity, particularly through the provision of fitness facilities (Fitness Australia 2012). According to the Australian 2010 Exercise Recreation and Sport Survey, fitness activities are the second most common sport or recreation activity undertaken by people aged 15+ years over the previous 12-month period (Australian Sports Commission 2010). In America, over 60% of those aged 6+ years participated in fitness sports (e.g. treadmill, free weights, boot camp, group exercise classes) in the previous 12 months and this is considered the most popular physical activity, according to the Physical Activity Council’s 2014 Participation Report (Physical Activity Council 2014).

Unfortunately, however, with all forms of physical activity, there is a risk of injury. Fitness activities performed in fitness facilities can have the potential to cause harm and injuries sustained by fitness activity participants can result in interruptions to their daily life, loss of income, temporary or permanent disability, or in severe cases, death (Andrew et al. 2014, Gray and Finch 2015). As the popularity of fitness facilities increases, so too does the risk of injury on a pure exposure basis.

Very few studies exist that investigate the injuries sustained during fitness activities, and most focus only on describing the types of injuries sustained (Thompson et al. 2001, Hayes 1985, Quatman et al. 2009, Salmon et al. 2000) or the rate/frequency with which they occur (Garrick et al. 1986, Jones et al. 2000, Kerr et al. 2010). Even fewer studies have given attention to understanding the specific causes of such injuries in the fitness centre context. The causes of injuries must first be identified to enable development and implementation of targeted prevention measures to reduce the future risk of such injuries.

Emergency department data which contains narrative details on the incidents and causal factors leading to injury is a potentially useful source of injury data (Gray and Finch 2015). Databases of this nature describe cases where the person injured has deemed the injury severe (or acute) enough for them to seek treatment at a hospital emergency department. This study aimed to examine the narrative text descriptions of emergency department presentations in Victoria, Australia, to identify the reported factors associated with the causes and/or mechanisms of injuries sustained at fitness facilities.

## Methods

Data were obtained from the Victorian Emergency Minimum Dataset (VEMD) through the Victorian Injury Surveillance Unit (VISU). Individual participant consent was not required as VISU has approval from the Human Research Ethics Committee at the Victorian Department of Health to provide summary data for this research. The VEMD, as managed by VISU, was queried to identify all injury-related emergency department presentations where the injury had occurred as a result of fitness-related activities over the entirety of the database (July 1999 to June 2013, inclusive).

The VEMD routinely collects data on injury presentations to all 39 Victorian public hospitals that have a 24-h emergency department, however not all of these contributed to the VEMD over the whole reporting period. Case selection from this database was based on targeted keyword text searching of 250-character narratives provided with each case. The purpose of the 250-character narrative is to provide more information about the specific circumstances surrounding the injury than is contained in the coded data. A keyword query for words/phrases relating to typical activities and equipment commonly used in fitness facilities was performed. This included terms such as treadmill, elliptical trainer, rowing machine, aerobics, weight training, barbell, dumbbell and cross training. Selected cases were restricted to people aged 15+ years, given that most fitness facilities enforce a minimum age requirement.

**Figure 1:**
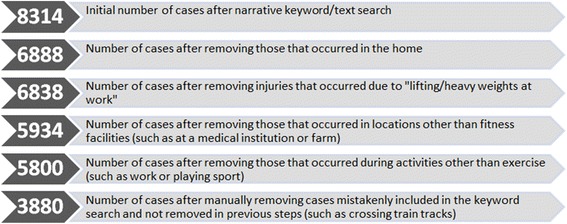
**The number of emergency department presentations retained after each associated data cleaning process.**

The extracted data required cleaning to remove irrelevant cases that were mistakenly selected by the keyword search (see Figure 1). Figure 1 shows the steps that were performed to condense and refine the dataset and includes examples of exclusions, so that only injuries that occurred within fitness facilities were included in the dataset to be analysed.

Using the information given in the narrative, each of the 3,880 extracted cases was manually reviewed and assigned 1 of 19 specific causes as shown in Table 1:

**Table 1:** **The 19 categories of causes assigned to ED presentations relating to injuries at fitness centres**

**Specific cause**	**Explanation**
*Trip/fall using static fitness equipment*	Any trip or fall when using non-motorised equipment (e.g. Swiss ball)
*Trip/fall using motorised/cardiovascular equipment*	Any trip or fall when using motorised equipment (e.g. treadmill)
*Trip/fall throughout facility (including group exercise)*	Any trip or fall when not using equipment (e.g. during aerobics class)
*Trip/fall in change rooms*	Any trip or fall that occurred in the change rooms (e.g. slip in shower)
*Awkward landing or twist during exercise*	Any mention of awkward landing or twist when exercising (e.g. rolled ankle in aerobics class)
*Caught in equipment*	Incidents where body parts have been caught in machines or equipment (e.g. finger in treadmill belt)
*Crushed by falling/dropped weights*	Dropping of heavy weights or objects onto body parts (e.g. dropped barbell)
*Crushed between weights/objects*	Body parts that have been crushed between heavy weights or objects (e.g. when placing plate weights on equipment)
*Hit/contact with equipment/wall*	Any case where individual has made contact with the wall or equipment (e.g. missed boxing bag and hit wall)
*Hit/contact with other person or self*	Any case where there has been contact with another or themselves resulting in injury (e.g. martial arts class and kicked by other participant)
*Crushed beneath equipment* (other than weights)	Any case where a body part has been crushed beneath equipment (e.g. equipment moved and landed on foot)
*Equipment malfunction/break*	Snapped cables, equipment has fallen apart or dislodged, or equipment has not worked as designed
*Moving equipment*	Any injury that occurred when moving equipment (e.g. injured back when moving treadmill)
*Overexertion/strenuous/unnatural movement*	Any mention of a sprain or strain, they felt something give way or ‘pop’
*Working/fitness instructor/demonstrator*	Any mention of injury occurring during work at fitness facility (e.g. class instructor fell from stage)
*Assault*	Any intentional injury caused by another
*Foreign body*	Any mention of foreign body in body cavity (e.g. rust from dumbbell in eye)
*Poisoning/allergy*	Any mention of individual attending a fitness facility and developing allergic reaction (e.g. used a piece of equipment and broke out in hives)
*Exact cause unknown*	Injury is known to have occurred at a fitness facility but exact cause not stated

Words in italics both in the above table and throughout the text are the cause categories.

Each of the *n* = 3,880 narratives was then manually reviewed and categorised into 1 of 29 specific activity groups that were subsequently collapsed into seven broad activity groups. These activity groups were determined by the information provided in the narrative about what fitness activity was being participated in at the time of injury and are shown in Table 2.

**Table 2:** **The seven broad activity groups encompassing each of the 29 specific activities**

**Broad activity group**	**Specific activities within the broad activity group**
Cardiovascular/motorised equipment	Treadmill, stepping machine, exercise bike, elliptical trainer and rowing machine
General fitness/aerobic exercises	Skipping, running, Swiss ball, boxing-related (non-class), stretching, step up, medicine ball, sit ups/abdominal exercises, push-ups and personal training session (activity unknown)
Group exercise class	Exercise group class, spin class
Resistance/weight training	General free weights (injury occurred by lifting weights but specific equipment not expanded on in text narrative), weight machine, barbell, dumbbell, bench press, squats, lunges or deadlifts, chin up/pull up and resistance band
Unspecified fitness activity	Any that is not specified and not included in other categories
Unspecified fitness equipment	Using equipment when exercising but does not specify which type
Working at facility	Working at facility

Cases that did not specify the exact cause of injury were excluded for the purposes of this study, therefore the final total number of cases analysed was 2,873. Cases were retained if it was clear that they were associated with fitness activities even when the specific fitness activity was not mentioned in the narrative.

Descriptive frequencies and cross-tabulations were performed in SPSS Version 21 to determine the leading fitness activities and causes that resulted in hospital ED treatment.

## Results

Over the 14 years of the VEMD, there were at least 2,873 cases associated with injuries that occurred at fitness facilities that specified the exact cause of the injury.

There were 807 cases (28.1%) where the narrative reported that an injury had occurred during exercise at a fitness facility but did not state the specific exercise being participated in at the time of injury. Similarly, there were 210 cases (7.3%) that stated an injury occurred when using, or due to, a piece of fitness equipment but did not further specify the exact type. For the purposes of Table 3, to determine the most common fitness activities associated with fitness injury-related ED presentations, the cases that did not specify the fitness activity or equipment were removed (*n* = 1,017, 35.4% of cases).

**Table 3:** **Frequency and proportion of fitness-related ED presentations according to common fitness activities and injury causes**

Fitness activity	*n*	Proportion of specified activities and equipment (*n* = 1,856)
General free weights - exercise unspecified	783	42.2%
Exercise group class	295	15.9%
Treadmill	167	9.0%
Boxing-related (non-class)	114	6.1%
Dumbbell	111	6.0%
Squats, lunges or deadlifts	56	3.0%
Bench press	46	2.5%
Exercise bike	45	2.4%
Swiss ball	29	1.6%
Barbell	28	1.5%
Specific cause	*n*	Proportion of specified causes (*n* = 2,873)
Overexertion/strenuous/unnatural movement	1,039	36.2%
Crushed by falling/dropped weights	468	16.3%
Trip/fall throughout facility (including group exercise)	360	12.5%
Awkward landing or twist during exercise	344	12.0%
Hit/contact with equipment/wall	275	9.6%
Trip/fall using motorised/cardiovascular equipment	148	5.2%
Trip/fall using static fitness equipment	68	2.4%
Caught in equipment	32	1.1%
Equipment malfunction/break	26	0.9%
Hit/contact with other person or self	26	0.9%

Note: Cases that did not state the specific fitness activity or equipment were excluded from this table (n = 1,017, 35.4% of cases).

Of the broad fitness activities that presented to EDs, resistance/weight training injuries accounted for 55.6% of specified activities, the highest of all activities. Of the specific fitness activities that presented to EDs, general free weights were the most common (42.2%). However when all free weight activities were combined (general free weights, dumbbell, squats, lunges and deadlifts, bench press, barbell), these accounted for 55.2% of all specified activity cases (see Table 3).

Amongst the specific causes, *overexertion/strenuous/unnatural movements* were associated with the highest number of fitness injury-related ED presentations (36.2%) followed by *crushed by falling/dropped weights* (16.3%), *trip/fall throughout facility* (12.5%) and *awkward landing or twist during exercise* (12.0%).

The six most common fitness activities and specific causes resulting in an ED presentation are summarised in Table 4. Over half of all fitness injury-related ED presentations were associated with the six most common fitness activities (53.1%). When the cases with unknown activities and equipment use at the time of injury were removed, however, 83.0% of the fitness injury-related ED presentations were associated with these top six activities. The majority of all fitness injury-related ED presentations were associated with the six most common causes (91.7%).

**Table 4:** **Frequency and proportion of fitness-related ED presentations for commonly reported fitness activities and causes**

	**Top 6 activities**																		**% of top 6 activities with that cause**
	**Treadmill**			**General free weights - exercise unspecified**			**Dumbbell**			**Exercise group class**			**Boxing-related(non-class)**			**Squats, lunges or deadlifts**			
Top 6 causes	***n***	**% of this activity**	**% of this cause**	***n***	**% of this activity**	**% of this cause**	***n***	**% of this activity**	**% of this cause**	***n***	**% of this activity**	**% of this cause**	***n***	**% of this activity**	**% of this cause**	***n***	**% of this activity**	**% of this cause**	
Overexertion/strenuous/unnatural movement	17	10.2	1.6	412	52.6	39.7	2	1.8	0.2	106	35.9	10.2	20	17.5	1.9	45	80.4	4.3	57.9
Crushed by falling/dropped weights	1	0.6	0.2	272	34.7	58.1	87	78.4	18.6	1	0.3	0.2	0	0.0	0.0	2	3.6	0.4	77.6
Trip/fall throughout facility (including group exercise)	0	0.0	0.0	9	1.1	2.5	3	2.7	0.8	84	28.5	23.3	11	9.6	3.1	3	5.4	0.8	30.6
Awkward landing or twist during exercise	13	7.8	3.8	28	3.6	8.1	0	0.0	0.0	76	25.8	22.1	15	13.2	4.4	4	7.1	1.2	39.5
Hit/contact with equipment/wall	2	1.2	0.7	31	4.0	11.3	11	9.9	4.0	13	4.4	4.7	54	47.4	19.6	1	1.8	0.4	40.7
Trip/fall using motorised/cardiovascular equipment	118	70.7	79.7	0	0.0	0.0	0	0.0	0.0	1	0.3	0.7	0	0.0	0.0	0	0.0	0.0	80.4
% of injuries associated with top 6 causes	90.4			96.0			92.8			95.3			87.7			98.2			

Table 4 shows that for both general free weights and exercise group classes, *overexertion/strenuous/unnatural movements* accounted for the highest proportion of injuries for each activity (52.6% and 35.9% of each activity, respectively). *Hit/contact with equipment/wall* was common for boxing-related activities (47.4%), and *falls from motorised equipment* were associated with the majority of treadmill injuries (70.7%). Dumbbell injuries were most commonly due to *weights falling or dropping* on the individual (78.4%).

Injuries due to *overexertion/strenuous/unnatural movements* most commonly occurred during general free weight activities (39.7% of injuries associated with this cause), and that was also the activity most often associated with *crushed by falling/dropped weights* (58.1% of injuries associated with this cause). When all free weight activities were combined, they accounted for 84.4% of crush injuries due to *falling or dropped weights*. More than half of trips/falls throughout the facility were during an unspecified activity (and provided no further information other than ‘trip/fall at facility’) (60.6%).

Eight percent of all presentations to EDs were considered severe enough to be subsequently hospitalised. The three most common causes that led to hospital admission, following ED presentation, were *crushed by falling/dropped weights*, *overexertion/strenuous/unnatural movement* and *trip/fall throughout facility*; however, these only accounted for 11.5%, 5.0% and 12.8% respectively of all cases associated with each of these causes. All free weight activities combined were the most common activities that led to hospitalisation (35.7% of hospitalisations), followed by treadmill and exercise group class (10% each).

## Discussion

The risk of injury from physical activity can interfere with the enjoyment of participation and reduce the long-term health benefits that physical activity can provide. Injuries can result in negative changes to daily activities, lost work time, poor quality of life, disability or in extreme cases death. To our knowledge, this study is the first to provide detailed information about the causes of fitness-related injuries on a large case series. Preventing injuries is therefore very important, but knowledge of how these injuries are occurring is required prior to designing and implementing injury prevention strategies. The findings from this study provide evidence for injury prevention strategy design and development and form a basis for risk management procedures that can lead to a reduction in incidents at fitness facilities.

Only three previous published studies have explored the causes of injuries associated with fitness activities (Gray and Finch 2015, Salmon et al. 2000, Kerr et al. 2010). One of these studies also used VEMD data and found that the majority of injuries associated with aerobics (or group exercise classes) were due to falls (Salmon et al. 2000). Assuming that the falls throughout the facility and awkward landings or twisting motions during exercise (which would likely then result in a fall) categories were combined, falls accounted for the majority of injuries associated with group exercise classes and is consistent with this VEMD study. Limitations of the VEMD aerobics study (Salmon et al. 2000) are that it was published more than 10 years ago and used only pre-coded data and so did not provide the same level of detailed cause information as in this study. Another study used both VEMD and Victorian Admitted Episodes Dataset (all hospital admissions in Victoria, Australia) data to determine the epidemiology of fitness-related injuries, provided an overview of the types and causes of such injuries (Gray and Finch 2015) and found that falls were most common during aerobics and when using other equipment; and the majority of resistance training injuries were associated with being hit or struck by weights or fellow exercisers.

A weight training study that used an American dataset similar to the VEMD found that injuries associated with free weights were more common than those associated with weight machines (Kerr et al. 2010). That study also found that crush injuries (by or between weights) was the most common, with overexertion as the second most common cause of injury. In our study, overexertion injuries were the most common; however, both were the main mechanisms of injury, which was very similar to this study. Whilst the study by Kerr et al (2010) reported injuries up to the year 2007, it used only pre-coded data, and the data is therefore less detailed than this study.

Haddon’s ten injury countermeasures is a good framework on which to base injury prevention strategies (McClure et al. 2004). Table 5 shows Haddon’s ten countermeasure strategies and how they could be used to reduce the likelihood or severity of injuries that occur in fitness facilities. This framework is used to make the recommendations for prevention outlined in Table 5 below.

**Table 5:** **Haddon’s 10 countermeasures with example strategies relevant to preventing injuries that occur within fitness facilities**

**Countermeasure**	**Example**
1. Prevent the creation of the hazard	Design building with high ceilings to allow for overhead lifts, add handrails alongside stairs and steps, hide all electrical cables beneath guards
2. Reduce the amount of the hazard	Limit the weight value that can be used without spotters
3. Prevent the release of a hazard that already exists	Install non-slip flooring, highlight changes in floor elevation (stairs/steps/ramps)
4. Modify the rate or spatial distribution of the hazard from its source	Install impact absorbing flooring
5. Separate, by time or space, the hazard from that which can be protected	Ensure adequate space around equipment (can be used as designed without contact with another walking past), separate activity areas
6. Separate the hazard and what is to be protected by a material barrier	Purchase only equipment that has protective guards around moving components; ensure mirrors do not touch the floor
7. Modify relevant basic qualities of the hazard	Applying contrast strips to all stairs, steps and changes in levels
8. Make what is to be protected more resistant to damage from the hazard	Recommend appropriate types and amounts of exercise to users
9. Move rapidly to detect and evaluate the damage that has occurred and counter its continuation and extension	Signpost broken equipment, mop up spilt liquids
10. Stabilise, repair and rehabilitate the damage or injured person	Train all staff in first aid and emergency procedures

Resistance/weight training injuries accounted for more than half of the presentations. Injuries associated with the use of a weight could often result from those unable to handle the amount of weight they are choosing or are required to lift (if weights were left by a previous user that required moving before the equipment could be used). Resistance/weight training activities are also technique orientated. Those who engage in such activities with incorrect technique are more vulnerable to both overexertion injuries and crush injuries, because incorrect technique can cause them to lose strength and a weight is dropped (Kerr et al. 2010, Hooper et al. 2014). Due to the nature of the activity and the equipment used, any injuries sustained during resistance/weight training activities would likely be acute and traumatic (Kerr et al. 2010), which could explain the high number of cases in our study of ED presentations. Acute, traumatic injuries are known to be more likely to require hospital treatment, rather than by allied health professionals or general practitioners (NSW Government 2014).

Injuries due to overexertion/unnatural/strenuous movements were common across many activities. This cause group included cases with narratives that mentioned ‘something gave way,’ they felt ‘something pop’ or something was strained or sprained. Injuries due to this category could be less preventable by facility design factors than other causes due to a person’s predisposition to injury (for example, body composition, tendinopathies and previous injury) (Taimela et al. 1990, Flynn et al. 2005) or if the individual was overexerting themselves or using incorrect technique (Hooper et al. 2014). It is recommended that facilities provide education regarding healthy amounts of exercise and how to perform each exercise with the correct technique. Facilities should also be supervised at all times by qualified staff to ensure members are using the correct technique, feeling supported and not engaging in dangerous behaviour (Fitness First Group 2014, American College of Sports Medicine 2012, Dietrich et al. 2014).

Injuries due to awkward landings or twisting motions may be difficult to prevent. In order to do this, fitness facilities should encourage proper footwear to ensure it provides the correct level of support and functionality (Haddon countermeasure 8), de-clutter or remove any trip hazards and, to a lesser extent, educate users to improve their technique.

Fitness facilities should provide proper storage for all loose equipment to ensure they remain tidy without equipment on the floor, which will help to prevent trips and falls throughout their facility (Haddon countermeasure 3). By appropriately highlighting changes in floor elevation (steps, ramps and edges) and providing adequate space around equipment so that others may navigate around it without interference when in use could also prevent trips and falls (Haddon countermeasures 7 and 4, respectively). Signs instructing users to wipe down equipment or the floor where sweat could cause a user to slip, as well as to put all equipment back in storage once finished, should also be displayed.

Trip and fall injuries from cardiovascular equipment could be prevented by instructing users to turn equipment off after use and ensure equipment is at a complete stop prior to getting on. Encouraging the attachment of the emergency brake cord when using particular equipment could reduce the severity of injury following a fall (such as a friction burn from a treadmill belt) (Haddon countermeasure 8).

Whilst Australia has no nationwide standards and guidelines for the fitness industry (Dietrich et al. 2014), the American College of Sports Medicine publishes and regularly updates their Health/Fitness Facility Standards and Guidelines. This book covers pre-activity screening, orientation, education, supervision, risk management and signage (American College of Sports Medicine 2012) and can be used as a good guide for Australian facilities to identify suitable strategies for removing injury causing risks and hazards.

Injury surveillance is crucial to the development of effective injury prevention strategies; however there is currently no comprehensive injury surveillance system for the fitness industry in Australia. Whilst it is possible that fitness facilities could maintain their own injury surveillance system, this data is not publically available and cannot be used to generate a cross-fitness industry profile. Therefore, the VEMD was queried as it is the only known publically available source of injuries associated with fitness facilities in Australia (Gray and Finch 2015).

A limitation of this study is that the VEMD may not be a representative sample of all fitness facility injury cases. Nevertheless, it has the potential to yield useful information about the causes of fitness-related injuries that cannot be obtained elsewhere and on a large number of cases. As the aim of this study was to identify the patterns of causes of fitness industry-related injuries, it did not matter that the coverage of the VEMD across all years varied. This study aimed to identify the main causes of injuries associated with particular fitness activities, rather than to determine the rate or frequency of injuries due to these. Case selection was based on targeted keyword searching of the 250 character narrative. Whilst every effort was made to allow for misspellings and variations (searched for ‘treadmill’ and ‘tredmill,’ for example), cases may not have been selected if a keyword was misspelt in the narrative and not accounted for during case selection. This could have possibly altered results; however, it is assumed that if any cases were not selected, it would likely have been a very small proportion of all fitness-related cases. Another limitation was that the VEMD was not set up for injury surveillance at fitness facilities nor was it set up to record the specific activity at the time of injury or the specific cause. Whilst the VEMD has its limitations, it can still provide useful information beyond the coded injury data regarding the circumstances of the injury.

## Conclusions

To our knowledge, this is the first study to report the specific causes of injuries across all major fitness activities. It provides relevant information from a large case series of injuries sustained by participants of fitness activities. In doing so, it describes the most common causes of injury and this information can be used to identify suitable prevention measures. Injuries occur to participants of all fitness activities, but the pattern of injury differs across activity types. Injuries due to overexertion were the most common overall as well as the main cause of injuries related to general free weight activities and group exercise classes. Crush injuries due to falling weights were common for all free weight activities. Falls were common during group exercise classes, throughout facilities and from cardiovascular equipment. This research provides the foundation for fitness activity-related injury prevention strategies to be developed, which should be tailored according to the type of fitness activity being undertaken.

## Abbreviations

ED: emergency department
VEMD: Victorian Emergency Minimum Dataset
VISU: Victorian Injury Surveillance Unit
